# Characterization of the Toxicological Impact of Heavy Metals on Human Health in Conjunction with Modern Analytical Methods

**DOI:** 10.3390/toxics10120716

**Published:** 2022-11-23

**Authors:** Dana Claudia Filipoiu, Simona Gabriela Bungau, Laura Endres, Paul Andrei Negru, Alexa Florina Bungau, Bianca Pasca, Andrei-Flavius Radu, Alexandra Georgiana Tarce, Mihaela Alexandra Bogdan, Tapan Behl, Aurelia Cristina Nechifor, Syed Shams ul Hassan, Delia Mirela Tit

**Affiliations:** 1 Doctoral School of Biological and Biomedical Sciences, University of Oradea, 410073 Oradea, Romania; 2 Department of Pharmacy, Faculty of Medicine and Pharmacy, University of Oradea, 410028 Oradea, Romania; 3 Department of Psycho-neurosciences and Recovery, Faculty of Medicine and Pharmacy, University of Oradea, 410073 Oradea, Romania; 4 Medicine Program of Study, Faculty of Medicine and Pharmacy, University of Oradea, 410073 Oradea, Romania; 5 Department of Pharmacology, School of Health Sciences & Technology (SoHST), University of Petroleum and Energy Studies, Bidholi 248007, India; 6 Analytical Chemistry and Environmental Engineering Department, Polytechnic University of Bucharest, 011061 Bucharest, Romania; 7 Shanghai Key Laboratory for Molecular Engineering of Chiral Drugs, School of Pharmacy, Shanghai Jiao Tong University, Shanghai 200240, China

**Keywords:** heavy metals, human health, tissue mineral analysis, mineralogram, environmental pollution, toxic effect, bibliometric analysis

## Abstract

Increased environmental pollution, urbanization, and a wide variety of anthropogenic activities have led to the release of toxic pollutants into the environment, including heavy metals (HMs). It has been found that increasing concentrations of HMs lead to toxicity, mineral imbalances, and serious diseases, which are occurring more and more frequently. Therefore, testing has become imperative to detect these deficiencies in a timely manner. The detection of traces of HMs, especially toxic ones, in human tissues, various biological fluids, or hair is a complex, high-precision analysis that enables early diagnosis, addressing people under constant stress or exposed to a toxic environment; the test also targets people who have died in suspicious circumstances. Tissue mineral analysis (TMA) determines the concentration of toxic minerals/metals at the intracellular level and can therefore determine correlations between measured concentrations and imbalances in the body. Framing the already-published information on the topic, this review aimed to explore the toxicity of HMs to human health, the harmful effects of their accumulation, the advantages vs. the disadvantages of choosing different biological fluids/tissues/organs necessary for the quantitative measurement of HM in the human body, as well as the choice of the optimal method, correlated with the purpose of the analysis.

## 1. Introduction

Any substance with the potential to produce a negative biological effect can be considered a toxic agent. There are several distinct and intricate classification systems for toxic substances, each based on different standards depending on the desired application. They are classified as pesticides, solvents, food additives, metals, and war gases based on their target purpose. Furthermore, classification based on the chemical structure of toxicants includes metals, non-metals, acids and bases, and organic toxicants, while analytical characterization divides them into volatile toxicants, extractives, metals, and metalloids. Therefore, metal toxicity is regularly encountered in a variety of forms, making the analytical assessment of heavy metals (HMs) highly important in the context of public health [1].

HMs are found naturally in the environment, in volcanic eruptions or soil, but they are also the result of various human activities, such as mining, automobile emissions, and industrial waste, etc. The term toxic metals is often synonymous with the term HMs, but there are also light metals which, through accumulation, or in certain forms or doses, become toxic [2]. They adversely affect living organisms, the environment and are non-biodegradable. The criteria for assigning the term HMs are as follows: atomic weight, atomic number, position in the periodic table, and specific gravity >5 g/cm^3^ (i.e., five times that of water) [3,4,5]. HMs are substances with high electrical conductivity, luster, malleability, and the property to split electrons, forming cations.

Concern over HMs contaminating the ecosystem has grown steadily, addressing the issues of environmental and global public health. As a consequence of their use increasing exponentially in a variety of manufacturing, agricultural, residential, and technical applications, human exposure has also increased significantly [6]. Based on the contamination of the air and water, or the accumulation of HMs in plants and meat, the population can be exposed to HMs directly by ingestion, inhalation, or direct skin contact. Furthermore, exposure to HM toxicity is more likely to occur in certain occupations [7].

**Figure 1:**
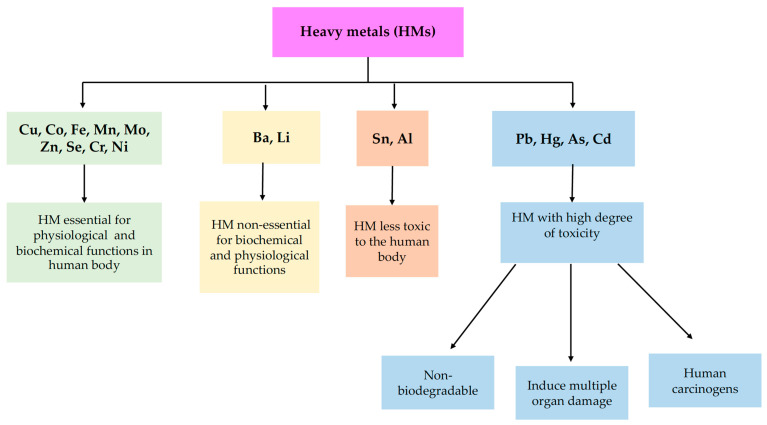
Classification of heavy metals based on their role in the human body.

Critical contaminants with the possibility of harming both human health and the ecological balance are non-essential metals. The toxicity manifested by non-essential HMs is different for each chemical element. Effects increase from a low level, as in the case of Barium (Ba), Lithium (Li), Aluminum (Al), and Tin (Sn) to elements with a high degree of toxicity, including Lead (Pb), Arsenic (As), Cadmium (Cd), and Mercury (Hg), responsible for numerous negative health consequences [8]. A classification system of HMs according to their role in the body is depicted in Figure 1.

HMs act as cofactors of the enzyme system, in biochemical or metabolic processes. Metal balance in the body is a widely acknowledged fact. When there is an imbalance, lowering or raising specific elements causes a decrease or increase in others. Such a disturbance of the balance can be the cause of many diseases/deficiencies [9].

Framing the already-published information in the literature on the topic (depicted in the bibliometric analysis), this paper aims to provide updated knowledge and a documented and validated database on the recognized toxicity of HMs, the harmful effects of their accumulation on human health, the benefits vs. disadvantages of choosing different biological fluids/tissues/organs for the identification and quantitative measurement of HMs in the human body, as well as on the choice of the optimal method of HMs analysis, depending on the purpose.

## 2. Criteria for Literature Selection

**Figure 2:**
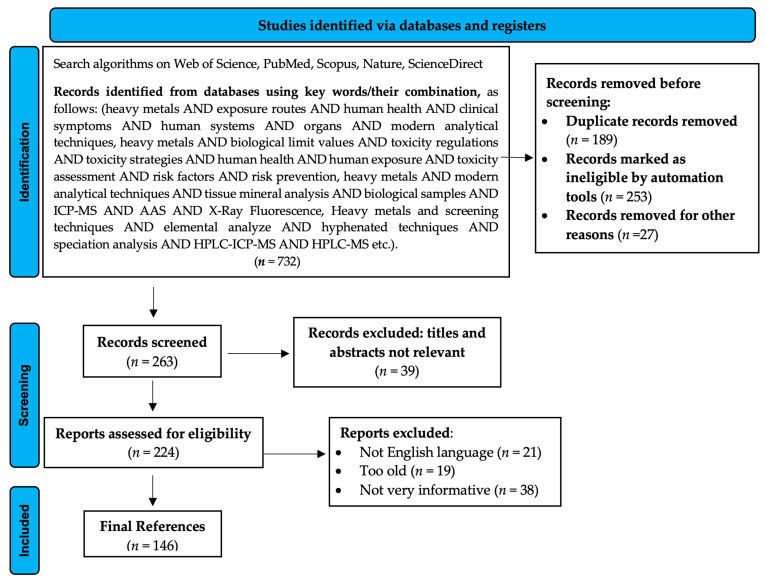
PRISMA 2020 flow diagram describing literature selection.

This paper reviews and centralizes publications on the toxicological impact of heavy metals on human health with an emphasis on modern analytical methods of detection and quantification, outlining the current state of knowledge in the field. In order to achieve this objective, advanced searches have been performed based on predefined algorithms using Boolean operators in large and scientifically validated databases (i.e., Web of Science, PubMed, Scopus, Nature, and ScienceDirect). Moreover, due to its export, filtering, analysis and interpretation facilities, Web of Science has provided the support for a bibliometric analysis of the data starting from a simple search. In total, 146 resources corresponded to the selection and evaluation processes and constituted a defining framework supporting the information in this analysis. The stages of bibliographic selection have been represented in Figure 2, which was realized according to Page et al.’s guidelines [10].

## 3. Bibliometric Analysis of the Topic in the Literature

### 3.1. Data Collection

**Figure 3:**
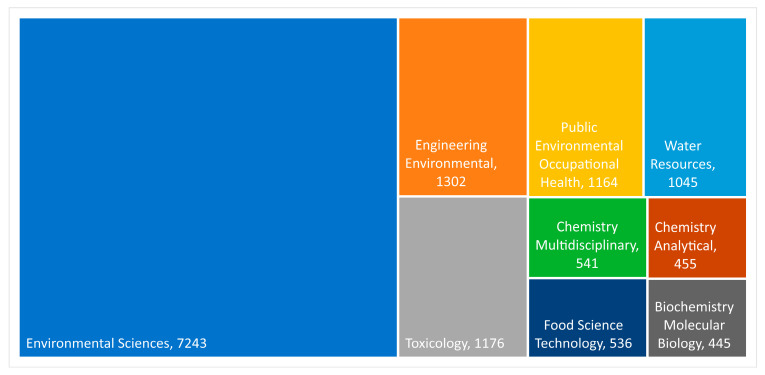
Treemap of WOS categories with over 400 documents. Under the category name, the number of articles is presented.

The Web of Science (WOS) database is one of the largest abstract and citation databases, and it was used to conduct the literature search. Using the following search parameters (TS = (heavy metal) AND TS = (human health)), 13,198 documents were identified. These documents are classified into the categories presented in Figure 3 (one document can fall into multiple categories).

Out of the 13,198 documents, only the documents written in English were included in this research (12,994). The leading countries in publishing papers, the relationship between them, the top-ten journals (according to the number of published articles), the network map of co-authorship, the top-ten most-cited articles, and the bubble map of the words with the highest occurrence and the average citations of these words are presented in the following section of the article.

### 3.2. Literature Results Obtained and Discussion

Pier, S. published the first document that fit the search parameters in 1975. The title of this paper is: “Role of heavy-metals in human health”; it was published in *Texas reports on biology and medicine*, and it has 21 citations. From 1975 to 2000, 159 articles were published. Then, from 2001 until 2022, the subject started to gain traction, and the number of articles published yearly started to increase steadily.

Figure 3 presents the number of articles published each year. Due to the low number of published articles before 1991, Figure 4 presents only the data from 1991 to 2021. The first year in which there were over 100 articles published was 2006.

The data was downloaded from the WoS export menu, using the following settings: “Full Record and Cited References.” Then, the bibliometric analysis was conducted using VOSviewer (CWTS, Leiden University, Leiden, The Netherlands).

#### 3.2.1. From 1975 to 2010

In this period, 1401 documents were published. Out of these documents, 1083 (77.30%) were articles, 280 (19.99%) were proceedings papers, and 164 were review articles (11.70%). The rest of the documents were classified into other categories. The most prolific countries were the United States, China, India, Spain, and England.

**Figure 5:**
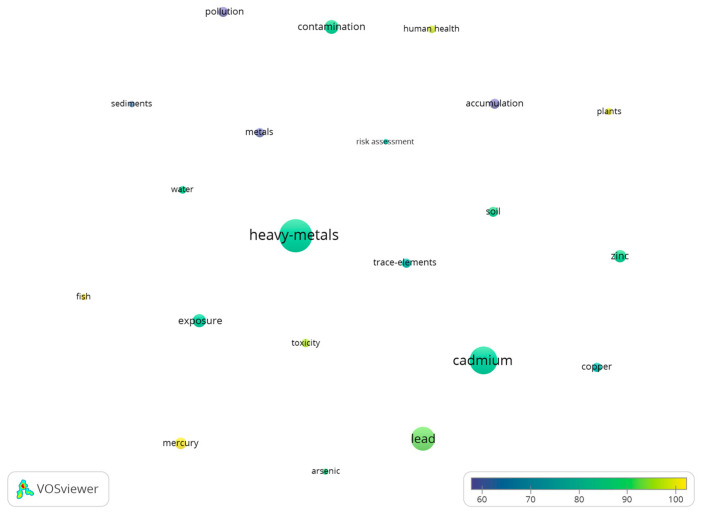
Bubble map of keywords co-occurrence from 1975 to 2000.

Figure 5 (21 items) shows the keywords co-occurrence map. The bubble size is directly proportional to the number of occurrences, the bubble color corresponds with the average citation/document that includes the keywords: if two bubbles are closer to each other, they have a higher co-occurrence level. Only words with a minimum occurrence of 50 are presented. Words with a high occurrence are presented in Table 1.

#### 3.2.2. From 2011 to Present

**Figure 6:**
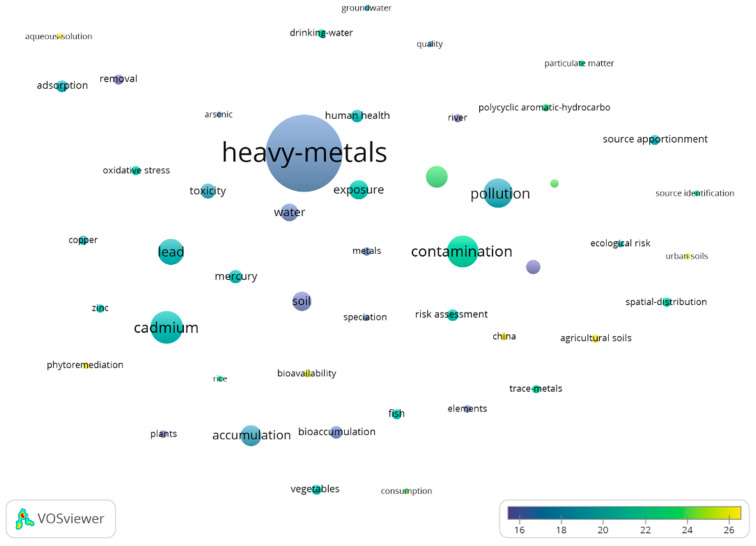
Bubble map of keywords co-occurrence, from 2011 to present.

A total of 11,593 documents were published in this period. Out of these documents, 9749 (85.01%) were articles, 1461 (12.60%) were review papers, and 469 (4.04%) were proceedings papers. The rest were classified into other categories. China was the most productive country, followed by the United States, India, Pakistan, and Iran. Figure 6 (52 items) shows the keywords co-occurrence map for this period. The minimum word occurrence was set to 300.

Table 1 compares the most-occurring keywords for both periods. Keywords, such as “heavy-metals” and “cadmium” had the highest occurrence for both periods. Words that represent HMs and were in the top-10 words based on occurrence, for the first period, were “cadmium,” “lead,” “zinc,” and mercury. For the second period, only “cadmium” and “lead” made it into the top-10 keywords list.

#### 3.2.3. Leading Countries in Publishing Papers Regarding Heavy Metal Detection

Authors from 167 countries published the 12,994 articles. Table 2 presents the top-10 countries according to the number of published articles, the number of citations, the average citation/document, and the total link strength (TLS—a score representing the strength of a country’s co-authorship with other countries). China had the highest number of published documents, with 3944 (30.35%), followed by the United States with 1346 (10.36%) publications. Countries that stood out because of the high average citations/document were the United States (39.33), Australia (34.49), Spain (31.27), and Pakistan (31.02)

**Figure 7:**
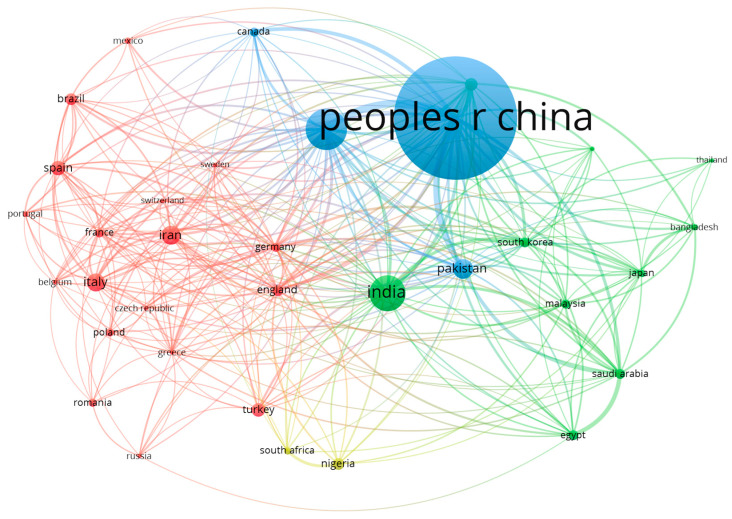
Bubble map of co-authorship by country (made with VOSviewer).

VOS viewer was used to create the network map of country co-authorship (Figure 7). Countries that collaborated in writing articles were grouped in clusters. The bubble size is directly proportional to the number of published documents, and the thickness of the line is directly proportional to the link strength (a measure of co-authorship between two countries). The bubble color indicates the clusters.

Countries with at least 100 published documents are presented. A total of 34 out of 167 countries met this threshold. The four clusters included the following countries:

- The red cluster includes Belgium, Brazil, Czech Republic, England, France, Germany, Greece, Iran, Italy, Mexico, Poland, Portugal, Romania, Russia, Spain, Sweden, Switzerland, and Turkey;
- The green cluster includes Australia, Bangladesh, Egypt, India, Japan, Malaysia, Saudi Arabia, South Korea, Taiwan, and Thailand;
- The blue cluster includes Canada, Pakistan, China, and the United States;
- The yellow cluster includes Nigeria and South Africa.

The two countries that collaborated the most were the United States and China, China and Pakistan, and China with Australia. Other countries that showed a strong collaboration were Saudi Arabia and Egypt.

**Figure 8:**
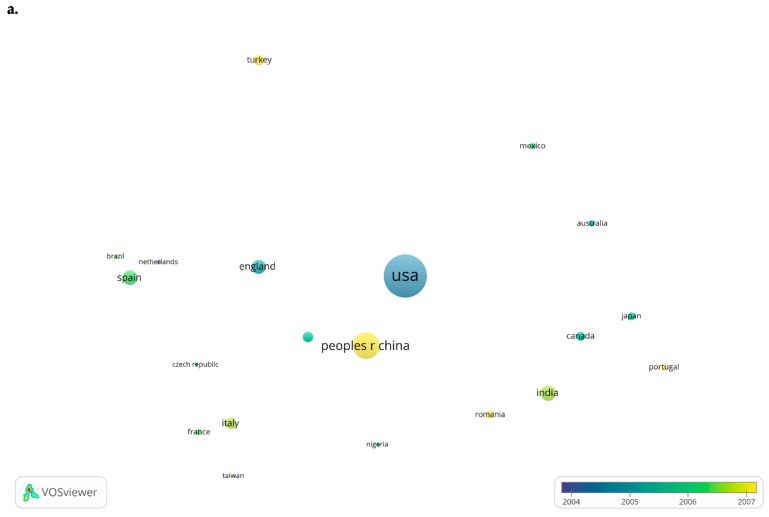

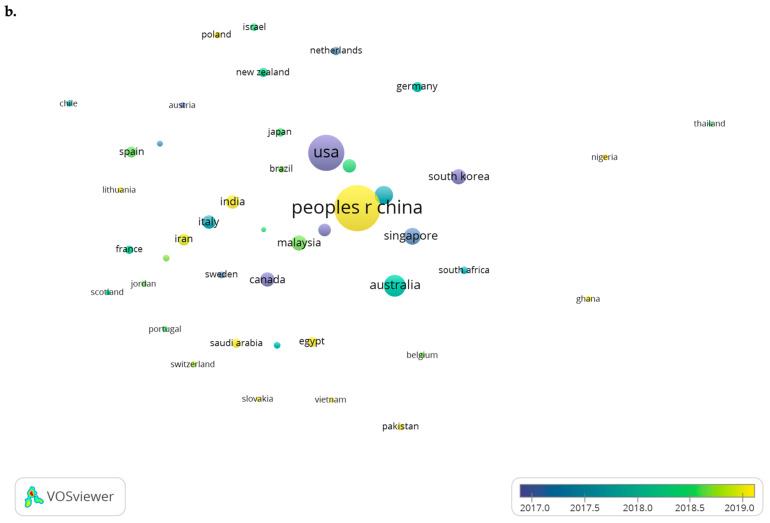
Most prolific countries: (**a**). In the period 1975–2010; and (**b**). In the period 2011 to present.

Furthermore, a bubble map representing the top-20 countries was created based on the number of documents to compare the two periods (Figure 8a,b). The bubble color indicates the average publication year of a country, and the bubble size is directly proportional to the number of published documents. Two bubbles are in closer proximity to each other if they collaborate often. For the first period, the United States was the major contributor in this field, but China overtook the United States in the second period regarding the number of published articles.

#### 3.2.4. Most Cited Articles and Leading Journals in Publishing Papers Regarding Heavy Metal Detection

The top-five most-cited articles are presented in Table 3. The most-cited article fitting the search parameters was “Hazards of HM contamination,” published in the *British Medical Bulletin* (IF 4.291) by Jarup L. in 2003, followed by the article published by Kampa M. in 2008 in *Environmental Pollution* (IF 8.071). The top-10 journals ranked by the number of published documents are presented in Table 4. The most prolific journal was *Environmental Science and Pollution Research*, with 750 documents published and an IF of 4.223. The journal *Science of the Total Environment* had the highest average citation/article, and the *Journal of Hazardous Material* had the highest IF of the presented journals (10.588).

**Figure 9:**
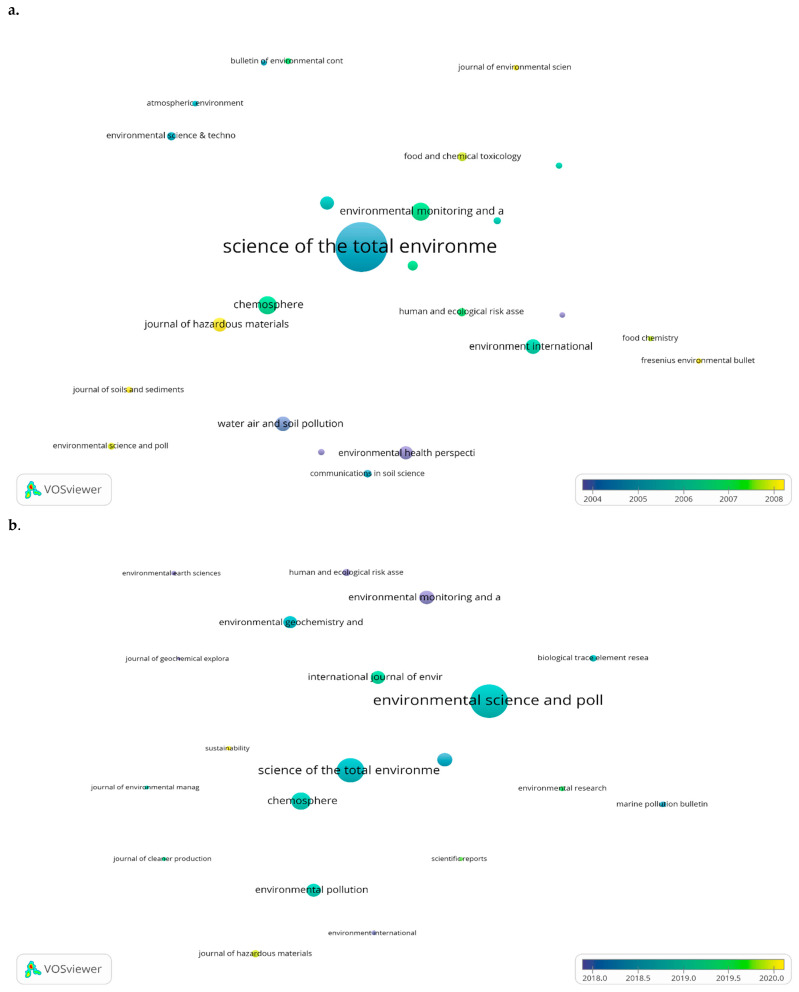
Most prolific journals: (**a**). In the period 1975–2010; and (**b**). In the period 2011 to present.

VOSviewer was used to create a bubble map of the sources that published the most documents in each period (Figure 9). The bubble size is directly proportional to the number of published documents. The bubble color represents the average publication year. For the 1975–2010 period, the most prolific journal was *Science of the Total Environment*, with an average publication year of 2004.82, followed by *Chemosphere* with an average publication year of 2006.62, and *Environmental Monitoring and Assessment* with an average publication year of 2006.78. The most prolific journal in the second period was *Environmental Science and Pollution Research* with an average publication year of 2018.78.

## 4. Exposure Routes to HMs and Toxicity Regulations

**Figure 10:**
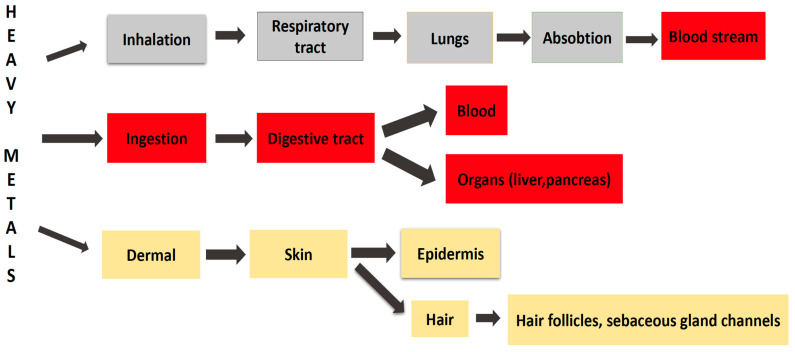
Exposure route to heavy metals.

Among the most toxic HMs (affecting human health), the following can be mentioned: As, Al, Hg, Pb, Ni, and Cd. Although they are extremely toxic, the population’s exposure to them is constantly increasing [15]. The HMs pathways into the human body are:

- ingestion (via food or water, reaching the bloodstream and various organs, such as the pancreas, and liver, etc., through the absorption process), as is the case for As, Pb, Hg, and Cd;
- inhalation (through the inhalation of air, vapors or aerosols, toxic metals enter the respiratory tract, reach the lungs and then the bloodstream), as is the case for Pb, Hg, and Al;
- and by dermal absorption for As, as is depicted in Figure 10 [16].

It is increasingly recognized that HMs have an increased affinity for certain target organs (i.e., liver, brain, kidneys, and the bones). The accumulation and toxic effect of HMs depends on several factors, such as concentration, chemical form, sex, age, and the time of exposure. Most of them, once ingested, are distributed through the bloodstream to various organs and tissues, where they cause damage in different systems and organs. HMs’ harmful action derives from stimulating the formation of free radicals and reactive oxygen derivatives, which cause lipid peroxidation and oxidative stress in the body [17,18]. Furthermore, it must be considered the excess of HMs in the human body which may lead to neurological disorders and adverse emotional changes [19,20,21]. Table 5 summarizes the toxic effects on some organs and systems.

Several institutions manage substance safety concerns and have developed legislation regulating HM-related issues. As a result of the harmful effects of HMs on human health, the Environmental Protection Agency (EPA) made several decisions, including decreasing the Acceptable Daily Intake (ADI) and establishing a Reference Dose (R*f*D), with an acceptable safety level on health, for developmental and non-carcinogenic effects [38].

The official estimations from Institutions, such as the EPA, Joint Food and Agriculture Organization and World Health Organization (FAO/WHO) Expert Committee on Food Additives (JECFA), Food and Drug Administration (FDA), and Agency for Toxic Substances and Disease Registry (ATSDR) that provide the minimum risk limits for HMs, are used to determine the daily maximum safe exposure levels. All the minimal risk values originate from dose-response measurements correlating chronic exposures with the impacts seen in individuals or animal models. The minimum risk values are all based on the prevention of adverse health consequences. Moreover, the chronic oral minimum risk values regulated by the agencies for adults are 2.14 μg/kg/day for inorganic As (JECFA), 1 μg/kg/day for Cd (EPA), 0.16 μg/kg/day for Pb (FDA), 0.3 μg/kg/day for methylHg (ATSDR), 1500 μg/kg/day for Cr (III), and 3 μg/kg/day for Cr (VI), both results being provided by the EPA [39]. The risk posed by HM pollution to human health, can be assessed by using different indices, such as the daily intake of metals (DIM), the transfer factor (TF), and the health risk index (HRI), etc. [40].

Due to the difficulty in quantifying the effects of HMs and due to the low (trace) concentrations in different biological matrices, biological monitoring (BM) of human exposure to HMs has become a real challenge. Therefore, HMs toxicity, related to their concentration and accumulation in the human body, over a long period, are triggering factors of various negative effects on human health [41].

The scientific literature includes experimental studies on the acute and subacute toxicity of HMs. The bacterial bioluminescent test of toxicity (BBTT) is utilized to assess the acute toxicity of several HMs. The toxicity assays involved the evaluation of the effective concentration levels over different periods and the relative molar toxicity estimates. An experimental study found that Hg was the most harmful of the group of studied HMs (Hg, Pb, Zn, Cu, Cd, As, Cr, Co, and Ni) to the bioindicator *Photobacterium phosphoreum*, while Ni has been found to be the least toxic [42]. Another study assessed the acute toxicity of Cu, Pb, and Hg on juvenile *Cyprinus carpio*, determining the concentration that is lethal to 50% of the species (LC50) after 96 h of exposure (0.30 ppm for Cu, 0.44 ppm for Pb, and 0.16 ppm for Hg) [43]. To study the impacts of acute toxicity on the survival rate and bioaccumulation of HMs in tissues, *Oreochromis* sp. was examined with increasing HM concentrations and times of exposure. The 96 h LC50 of Cu, Zn, and Cd were found to be 0.45, 2.1, and 0.7 mg $L^{-1},\;\mathrm{respectively}$. Inductively coupled plasma optical emission spectrometry (ICP-OES) was used to examine fish tissues. A higher concentration of hazardous metals was associated with considerably higher tilapia fish mortality. The results showed that, in these experimental conditions, Cu was more toxic than Zn or Cd [44]. Furthermore, when HMs are present in the environment (water, soil, and food crops) at levels higher than the reference values, there is a significant risk to human health. The well water standard regulation’s reference values are 0.07 µg $L^{-1}$ for Cd, 2.52 µg $L^{-1}$ for Cu, 0.39 µg $L^{-1}$ for Pb, and 1.36 µg $L^{-1}$ for As. The mean maximum allowable levels (MALs) for the presence of HMs in soils are 13.7 mg ${\mathrm{kg}}^{-1}$ for As, 28.5 mg ${\mathrm{kg}}^{-1}$ for Pb, 0.18 mg ${\mathrm{kg}}^{-1}$ for Cd, and 32. 8 mg ${\mathrm{kg}}^{-1}$ for Cu. Moreover, the MALs for the presence of As, Pb, Cd, and Cu in vegetables and cereals are 0.1 mg ${\mathrm{kg}}^{-1}$, 0.2 mg ${\mathrm{kg}}^{-1}$, 0.125 mg ${\mathrm{kg}}^{-1}$, and 10 mg ${\mathrm{kg}}^{-1}\;$[45], respectively.

The most commonly used methods for exposure assessment include sample selection and preparation steps, followed by sample digestion prior to qualitative and quantitative analysis. The properties of HMs make atomic absorption spectroscopy (AAS) a frequently used method due to its high sensitivity and specificity. The evaluation of the relevant certified reference materials is used to verify the validity of the analytical processes [46].

The basic standard necessary to protect individuals from risks to their health and safety inflicted or anticipated to be caused by the presence of HMs at work or as a result of any occupational activity using chemical agents is regulated by law. Furthermore, it is mandatory to respect the biological limit values tolerable by employees, with the concentrations of chemical agents kept as low as possible. Table 6 presents the binding biological limit values (BBLVs), including HMs, established by the Scientific Committee for Occupational Exposure Limits (SCOEL), Chemical Agents Directive (CAD) 98/24/EC establishing the biological limit value at the European Union level and national regulations [47].

According to Omrane et al., three strategies have been applied for the assessment of occupational exposure to HMs in order to correlate with the models’ predictions, including mathematical algorithms to estimate analytes’ concentrations, the determination of indoor HM concentrations, and biological testing of HMs in the urine of consenting adults. Moreover, in this experimental study, inhalation was the principal exposure route based on the selection criteria. The concentration of HMs is predicted by using a variety of methods (i.e., the Eddy Diffusion Turbulent model, the Well Mixed Box, and the Near Field and Far Field) [48].

The measurement of a chemical agent or its metabolites in a biological sample is one method of determining exposure through BM (usually blood, urine or breath). The benefit of BM is that it incorporates all exposure pathways and sources [49,50,51,52]. BM can be separated into exposure assessment and effect observation, for which internal dose and effect markers are being used. In the context of environmental health, biomarkers mostly refer to measurements used for risk evaluation or diagnosis. The phrase is now being used to describe BM measurements in occupational medicine or industrial health, too. The monitoring of exposure and effects correlate with the indicators of exposure and effects [53].

In the domain of environmental health, a number of biological methods and biomarkers are helpful for determining the risk of lead exposure. The most popular biomarker for lead exposure is blood lead. While the significant effects of lead on bone marrow can be considered an effect biomarker, this indicator assesses body burden, soft tissue lead, and absorbed dosages of lead. The interaction of lead with certain enzymes involved in heme production is the primary cause of lead’s effects in the bone marrow [53].

The suppression of delta-aminolevulinic acid dehydratase (ALAD) and changes in the quantities of certain metabolites, such as delta-aminolevulinic acid in urine (ALA-U), zinc protoporphyrin (ZP) in blood, delta-aminolevulinic acid in plasma (ALA-P), delta-aminolevulinic acid in blood (ALA-B), and coproporphyrin in urine (CP), are the key biomarkers of impact. The aforementioned markers do not, however, all accurately reflect the dose and the internal dose/effect correlation [54].

The heme pathway’s rate-limiting enzyme, ALA synthetase (ALAS), creates ALA in the mitochondria from succinyl-CoA and glycine. Lead exposure causes a reduced ALAD activity, and indirect ALAS induction caused by negative feedback control, resulting in an increase in ALA in different tissues and plasma and an increase in ALA excretion in urine. Although ALA in urine (ALA-U) has been suggested as a biomarker of lead toxicity, ALA in plasma or blood indicates the impact of lead on bone marrow more specifically. In a wide spectrum of Pb-B values, an exponential link between plasma and urine ALA was noticed [55].

The proportion of biological anomalies may be indicated by the free erythrocyte protoporphyrin (FEP) values. Microcytic anemia is linked to noticeably high BLLs. The importance of FEP measurement may be muddled by iron deficiency, which is also linked to anemia [56].

The levels of zinc protoporphyrin (ZPP) are frequently applied as indicators for lead toxicity. The other essential enzyme that facilitates the inclusion of iron into protoporphyrin IX is ferrochelatase. However, this enzyme is suppressed, and the route is blocked in Pb toxicity conditions. Additionally, if there is not enough iron available, Zn is used in place of Fe, which raises the concentrations of ZPP. These increases do not manifest in the blood until Pb levels approach 35 μg/dL, which limits this modification as a significant diagnostic characteristic [57].

## 5. The Importance of Tissue Mineral Analysis

In the United States in the 1970s, the first mineralograms were made by mass spectrometry, a technique used at the time by NASA, to analyze the moon rocks, as well as to analyze the metals from the spacecraft’s shell. Starting from here, this technique was also used in medicine, as an analysis for the determination of the metal content in animal fur, as well as in the human hair [58].The idea of mineral analysis prompted several American researchers, for example: Dr. George Watson (Nobel Prize), Dr. Clivet, Dr. David Watts, Dr. Wilson, and Dr. Paul Eck, to study numerous cases for more than 40 years, and to conclude that the mineral levels from the hair, correlated well with the mineral level from the tissues. In 1980, the EPA recognized the tissue mineral analysis (TMA), as a scientific method of determining toxic substances, minerals, metals from the human body.

Research on TMA has shown that it is a very advanced and modern method in medicine, which shows the relationship between human health and the concentration of various HMs/nutrients. We can find information about the time of exposure, absorption, and the distribution of all toxic substances in human tissues/organs. After absorption, HMs pass into the bloodstream and are stored into the target organs/tissues, which shows that the blood does not reflect an older exposure, this fact being found in the studies on children previously exposed to Pb. In conclusion, we can say TMA is probably the most important barometer regarding the evaluation of HMs on human health [19]. With this method, in a single analysis, the most important 36 minerals from the body can be determined, of which 30 are essential and 6 are toxic minerals (HMs), in extremely low quantities—parts per million (ppm) or even per billion.

The human body’s organic structure is made up of 80% water and 19% minerals. Minerals play a very important role in the normal functioning of the body, by controlling certain biochemical reactions, stabilizing components of proteins and enzymes, functioning as mineral cofactors for many enzymes and antioxidant molecules [59]. The enzyme with a mineral cofactor (a bivalent ion of Fe, Zn, Mn, and Co) forms an active configuration. HMs, such as Pb, Hg, Cr, As, and Al, can interfere with enzymatic reactions and disrupt their enzymatic activity because of their affinity for thiol groups (-SH) from the enzyme [60].

As was mentioned before, using TMA, the concentrations of HMs and minerals are determined, whose values provide information at the intracellular level, enabling us to identify the tendency towards functional imbalances, chronic intoxications, and concentrations, which cannot be determined by blood or urine analysis (this analysis provides information only at the extracellular level). In other words, we can say that this analysis shows the stress response phase, the mineral requirement of each human organism, the HM amount, carbohydrate metabolism, and the tissue hormone efficiency, etc. Thus it can be stated that TMA with an individualized clinical examination, allows an accurate diagnosis, the establishment of a nutritional strategy, the amelioration of various mineral and hormonal imbalances, and can even identify possible HM intoxication [61]. In the last years, TMA has greatly improved through Hair Analysis (HMS—Hair Metabolic System), which is a more representative method in terms of prevention for preventive diagnosis. Therefore by using HMS, the concentrations of different minerals/HMs can be determined, for up to 3 months or even more, and can be used for individual personalized treatments (in HM intoxication: the chelation therapy or homoeopath treatment; in the case of mineral deficiencies by taking dietary supplements or vitamins) [62]. The continuous development of TMA has led to the discovery of many correlations between disease and emotions. It is well known, that most diseases have an emotional character, and emotions are enchained with the biochemical changes in the body. Emotional and mental factors can lead to increased excretion and absorption of minerals/HMs by the human body. From this, we deduce that strong emotional reactions will produce nutritional, hormonal, neurological, and metabolic changes, which may be quantified and TMA can be that barometer/instrument for assessing the psycho–somatic and somatic–psychic relationship [19].

## 6. Human Biomarkers—An Assessing Health Status Factor

One of the assessment methods used in the determination of HM toxicity to human health, is human biomarkers. The low concentration of HM in biological matrices, the quantification of the contributions of sources of exposure, environmental and genetic factors, have led to the difficulty of monitoring human exposure. However, the most widely used human biomarkers in toxicological study remain: blood, urine, nails, and hair [63]. Being a noninvasive matrix, the hair and nails are most-often used as a representative human biomarker in long-term exposures to HMs [64,65]. On the other hand, biomarkers, such as the blood and organ tissues, can decrease the rate of participation in the population, requiring invasive sampling. The majority of HMs have a relativity short lifetime, which makes their blood and urine dosing a standard only for short-term exposures, for example, 2–3 h or 3–4 days (acute exposure) [66]. Some of them, the most toxic HMs, such as Pb or Cd, remain in the blood and tissues for a long time. A quantitative assessment of them can only be made if the bioaccumulation method is well known.

### 6.1. Hair, Nails, Urine, Blood, or Vitreous Humor Analysis

The utility of studying and measuring the concentrations of HMs in hair, nails, various biological fluids (blood, urine, and vitreous humor), and some organs or tissues, has become increasingly vital, because all these biomarkers are a perfect barometer for the disturbances that occur in the human body. The determination of HMs/trace elements from the human blood or urine, is a traditional/classic procedure, which shows the concentration of HMs/trace elements at the present time, but the action of external factors and bio-kinetics, lead to fluctuations in the HMs/trace element concentrations in the urine and blood. The hair and nails are considered to be final metabolic products, which, compared to other human body compartments, provide clear and precise information about any trace elements, because we presume that they can incorporate very easily any trace elements (toxic or not) during the growth process, due to a complex structure called amino acids, made from sulfur and fibrous proteins [63]. In addition to the hair and nails, in some cases, hard tissues can be used as a biomarker, depending on the HM which must be determined [67]. All this HM accumulation in human body can induce certain diseases, intoxications or imbalances in the human body [68].

#### 6.1.1. Human Hair and the Trace Elements Stored in It

Human hair is made of approximately 80% protein, 10–15% water, 5–10% pigments, minerals, lipids, and the mineral content is 0.25–0.95% [69]. The scalp is usually made up of about 100,000 hairs, of which about 10% is at rest. The growth rate of the hair is generally 0.3 mm/day, which means about 1 cm/month. The hair follicle is strongly vascularized, due to the blood, which bathes it and is a very good transport medium for toxic and trace elements. As the hair grows, it suffers a keratinization process (hardening) and during this process, it incorporates all the traces of toxic and essential elements into the protein structure [68]. In 1929, the hair scalp was used for the first time, to assess the human systemic level of elements [70]. Following this analysis, the researchers established different correlations between the essential/toxic elements and different diseases, nutritional status, and metabolic disorders [71,72].

Even nowadays, the hair remains the most important biological matrix for health assessment, and for identifying possible intoxications of various etiologies (HMs, drugs, and pesticides), as well as mineral imbalances in the human body. Compared to other tissues/body fluids, such as the blood or urine, it was found that the concentration of HMs from the hair is in direct correlation with various diseases. HMS provides information about the history of long-term exposure, compared to other traditional analysis, which shows the toxic elements in the initial phase. The hair analysis is a non-invasive method, which does not require a large amount of biological material, is simple and straightforward. Hair has long been used, both in animal and human studies, to highlight the human body’s exposure to HMs. In this way, analyses can be performed on samples that are hundreds of years old [73].

Difficulties arise due the lack of well-defined reference areas of concentration. This is a consequence of the huge difference in the element’s levels of concentrations, depending on the region, sex, age, hair color, and lifestyle. Other external factors/sources (hair dyeing, use of cosmetics to treat hair, hair washing, and bleaching), may also influence the correct interpretation of the results [74]. The concentration of toxic elements in human hair, may also be affected by seasonal variations or the effect of synergism and antagonism [75].

#### 6.1.2. Finger- and Toenails

The most attractive and interesting samples sources are the nails and human hair, because they are very easy to collect, store and transport, unlike other biological fluids, such as the blood, urine, and vitreous humor, which can be easily contaminated if the collection and storage procedures are not followed correctly. These metabolic products have fairly consistent content of HMs due to the growth process [22].

The human nail is a thin, horny plate, which grows on the upper part of the phalanx of the fingernail and toes, having an important sensory and protective function. It is made of a strong protein material, called keratin, that incorporates all the essential trace elements or toxic elements. Like any other part of the human body, they are exposed to various environmental and occupational factors or to imbalances in the human body. Toenails and fingernails, are considered to be very good biomarkers for tracking accumulation of various toxic elements [76]. The trace elements and HMs are collected and stored for a very long period, which allows nail analysis, along with hair analysis, to bring valuable information about possible intoxications or diseases that exist in the body or favor the appearance of various diseases [77]. On average, the fingernail growth rate is about 3–5 mm/month, and the toenails increase by about 1.6 mm/month. This rate of nail growth depends on sex, age, health conditions, and metabolic rate, etc. However, toenails are more protected than fingernails from any possible external factors. Because nails incorporate any trace elements very easily in high quantity, they are considered to be an important barometer for long-term exposures, for all kinds of toxic elements [61].

Nails are made of a rich layer of keratin, which easily incorporates any trace elements, which makes by them an important source of vital information to the researcher. Along with hair, they are the most suitable sample for a long-term assessment of trace elements, which can cause intoxications or imbalances in the body. The taken sample does not require special conditions for collection, storage, and transport. In the case of some trace elements/toxins, it does not always provide all the necessary information. There may be differences in toenail analysis compared to the analysis of the fingernails, because the toenails are more protected from the action of various toxic factors and are washed less often than the hands. The growth of the toenails is slower than that of the fingernails, so the accumulation of HM is slower, on average about 2–12 months after exposure. Nails can be very easily contaminated due to the use of nail polish or some medications. Sometimes the amount of nails collected is quite small, which makes the limit of detection lower [76].

#### 6.1.3. Vitreous Humor and Epithelial Tissues (Human Eyes)

The toxicity of HMs is explained by the interaction between the HM ion and the specific target protein. This action changes the function and structure of the protein. The cells involved in the transport of HMs are very susceptible to their toxic effects [78,79]. The retinal epithelium is a chelating metal tissue, with a high affinity for HMs, due to melanin (the retinal tissue pigment), which forms bonds with HMs. Results have shown that Pb may accumulate in the retina and choroid [80].

#### 6.1.4. Blood and Urine

There are other biological matrices that can provide important information about the accumulation of HMs, because some of the trace elements are not found in the blood (note: it is preferable to determine the inorganic forms of Hg and As from blood). Some HMs can be stored in various tissues, such as the kidney, liver, brain, bones, and lungs, which are considered target organs, where HMs can cause a lot of injuries without increasing the values in the blood. The HMs remain only for a short time in the blood (blood has the role of transporting nutrients from one tissue to another), where they are fixed in the tissues or organs, so their values from blood are relevant for a short period of time. As a biological matrix for the determination of Pb, the blood is preferred, because it shows a better correlation with some diseases. In addition, the determination of MeHg salts is recommended from the blood. Blood and urine show us the amount of HMs at the present time, or better said, at the time of exposure ±2 days and not the cumulative degree of exposure.

The HM determination from the blood, provides information about recent exposure (hours or days). Blood determination is an invasive method, and the blood levels are transient, independent of those stored in human tissues (the toxic minerals are purified quickly) and vary from one component to other (e.g. the serum, and plasma, etc.) In the case of urine, it can be seen, that HMs are excreted at the time of sampling and provide information about relatively recent exposure (days or weeks), which makes this determination suitable for measuring acute exposure.

### 6.2. Different Tissues and Organs

#### 6.2.1. Teeth and Bones

Along with other biological fluids and tissues, the teeth and bones can be considered an important compartment in terms of exposure to HMs on a large scale [81]. It is well known that the teeth and bones are calcified tissues, which have as their main component hydroxyapatite. Although calcified (hard) tissues are chemically stable, it is presumed that some HMs/trace elements can accumulate in the skeletal structure, by displacing Ca^2+^ from hydroxyapatite, which makes the teeth and bones a bioindicator/biomarker for monitoring the accumulation of HMs in the human body [82]. From a morphological point of view, there are two types of bone tissues: spongy and compact. Spongy bone can provide information about a relative recent exposure to HMs, while within the compact bone, HMs can accumulate for a much longer period. During the formation of tooth tissues, there is no release of mineral substances, thus the teeth can be considered a permanent and stable recording in terms of exposure to HMs over a longer period [81,83]. In the case of bone tissues, in pathological and physiological conditions, a release and fluctuation of HMs/minerals from the bone tissues in the blood was found. Deciduous teeth cannot be considered a bioindicator of HM exposure, because they have a short lifespan, and later they are replaced by permanent teeth. Permanent teeth, especially the ‘dentin’ (which does not suffer any turnover mineral phase), can provide information about HM accumulation, but the tooth enamel, which is constantly in direct contact with various factors within the oral cavity (drugs, caries, smoke, food, and medications), can modify the mineral composition of the surface layers of the tooth, which may affect their credibility as a bioindicator [82]. Teeth are considered to be easy-to-extract biological tissues, with a low rate of pollutant removal [84]. As mentioned earlier, HMs, once ingested and absorbed, are distributed in target organs and those in large quantities, with a long half-life, are stored in the human skeleton, which is an ideal long-term storage place [85]. With aging, senescent cells begin to accumulate in organs and tissues. They can cause inflammatory reactions, and decreased vitality of cells and tissues. Thus, the bones may be affected by the distribution of HMs in the aging process. Human bone, being a hard, calcified tissue during growth, incorporates HMs, similar to the nails and hair. Compared to the blood, the skeletal system incorporates and retains HMs for a very long time, and with aging, the storage capacity can change.

Many articles have shown that there are no significant differences in HM concentrations between different bones (e.g., the femur, and tibia) [72]. However, a potential factor of influence is the place of sampling. Another disadvantage of bone sampling is the amount of the sample; the human bone is not always accessible for measurement or sampling [71].

#### 6.2.2. Tissues and Organs

It is well known that HMs have an increased affinity for certain organs (target organs) and systems. Both organic and inorganic HMs derivatives are toxic, but inorganic derivatives are the most toxic (creating irreversible injuries); they penetrate the fat-rich tissues, such as the brain, and liver, due to their increased fat solubility (i.e., Pb penetrates the bones and teeth 80–95%; the liver, lung and brain as soft tissues [22]; Hg (depending on the organic form) targets the lung, kidney (40–60%), heart, liver, brain, and tissues [86]; As targets the blood, lung, heart, kidney, and neurological systems; Al targets mainly the brain and bones, lung, muscle, heart, and spleen).

As previously stated, the determination of HMs from various organs and tissues, is extremely important, but too inaccessible (organs or tissues sampling may be performed only in the case of surgery or forensic cases/necropsy). As can be expected, any determination of HMs from various biological fluids, organs, or tissues, has advantages and disadvantages. In the following, the advantages and disadvantages of the biological matrix that contributes to the determination of HMs, are described and summarized in Table 7.

## 7. Modern Analytical Methods of Determination

**Figure 11:**
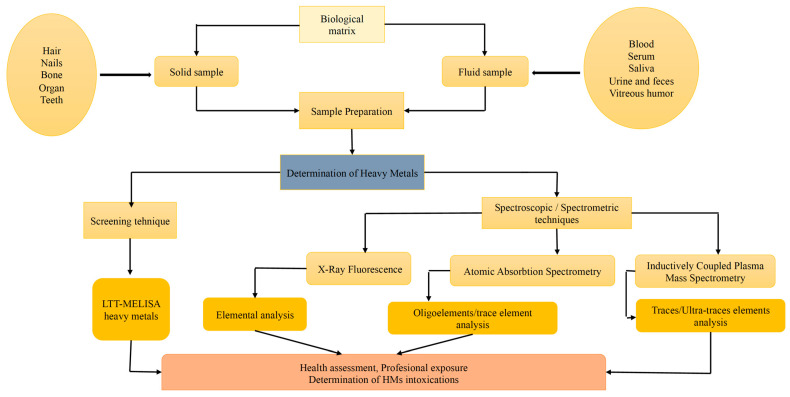
Biological matrix, Sample Preparation and Modern Analytical techniques in HM determination.

At the present time, the chemistry of HMs or HM traces, present or accumulated in the human body over time, can be determined through the continuous improvement of various specific, modern, selective, and highly sensitive analyses of several types of samples [100]. One of the most widespread analysis techniques, both in the field of elemental and molecular analysis, is spectrometry. When precise determinations are sought, it is imperative to apply spectrometric techniques, which cover an extremely wide field, and have precision and accuracy in determinations, which make the use of these techniques more than necessary [101]. Some of the most commonly used techniques are: Lymphoblastic Transformation Test (LTT)-HMs test, X-ray Fluorescence, AAS, Inductively Coupled Plasma Mass Spectrometry (ICP-MS) or Inductively Coupled Plasma-Atomic Emission Spectrometry (ICP-AES), and the reason behind these choices is mentioned later. The last three spectrometric techniques are the most often used to determine traces of heavy metals in various biological matrices (Figure 11) [102,103].

### 7.1. Lymphoblastic Transformation Test—HMs

One of the routine tests when it comes to human body sensitivity to HMs, is the Patch Test, or rather the Epicutaneous Patch Test. However, this test has some disadvantages: it can be false-negative or false-positive, it is subjectively assessed, it has low reproducibility, it can cause exacerbated irritation, and it is generally used to determine dermal allergens, the most common ones being Ni and Co [104,105].

The in vivo lymphocyte transformation test (LTT) is an alternative to the epicutaneous test (Patch test), which in turn is optimized by the LTT-MELISA^®^ (Memory Lymphocyte Immune Stimulation Assay) test-method accredited in Germany since 2001. LTT-MELISA^®^ is a test with high specificity, reproducibility, sensitivity, and reliability for the detection of sensitivity in patients with metal sensitivity [106].

The LTT is a test that shows the degree of proliferation (reactivity) of T lymphocytes, after exposure to various allergens (HM in this case). LTT-HM is the validated test for various allergens: respiratory, medicinal, and food; it does not produce sensitization, has an increased reproducibility, and provides objective results. It is indicated in a case of monitoring symptomatic persons, who are periodically exposed to the action of HMs. Thus, it was found that in the case of monitoring a patient exposed to the action of HMs, after avoiding exposure of the patient for a long time, the lymphocyte reactivity had normalized (LTT-negative) [107]. The test is performed through venous blood sampling, and it allows us to detect concentrations of HMs in saline solutions, such as Al, Gold (Au), Silver (Ag), Beryllium (Be), Cd, Co, Cr, Cu, Gallium (Ga), Indium (In), Iridium (Ir), Mo, Ni, Palladium (Pd), Pb, Platinum (Pt), Ruthenium (Ru), Sn, Titanium (Ti) and oxides of titanium (TiO_2_), Vanadium (V), as well as Hg compounds (i.e., ethyl Hg, MeHg, PhenylHg (PhHg), and Hg chloride (HgCl_2_) [108].

The MELISA^®^LTT-test is important in clinical relevance in the case of exposed patients, for those who are about to undergo surgery (i.e., prosthesis or dental work), or for those who suffer from chronic infections, fatigue or various autoimmune diseases, whose aim is to detect and monitor the action of HMs [108]. Research on the reliability of MELISA testing is inconsistent [109]. Some claim the MELISA test is reliable and clinically helpful [107,108,109,110]. However, other studies claim that MELISA testing is non-specific, therefore it has little clinical use [111,112].

### 7.2. X-ray Fluorescence

X-ray Fluorescence Spectrometry (X-ray Fluorescence) is a primary analytical technique, one of the oldest nuclear techniques that relies on the interaction of X-rays with a material, in order to determine its elemental composition [113]. X-ray fluorescence allows the determination of each individual element with high selectivity. This technique is suitable for flat, homogeneous, and very thin samples, as it has a low energy, which is easily absorbed by the sample matrix [60]. It is also suitable for the determination of heavy metals with Z > 18, from various biological materials. It is possible that with this method, a low concentration of elements can be found, which should be analyzed by other analytical techniques [114]. Compared to other analytical methods, this non-invasive technique does not require sample preparation. Due to the extremely penetrating nature of these X-rays, since their discovery, they have been used for non-invasive” observation, for the diagnostic purposes of various biological samples (i.e., synovial fluid, blood, plasma, and bones), environmental samples (i.e., soil, water, and oil), as well as food samples (i.e., milk, wine, and beer) [87]. It is a fast, simple, relatively low-cost method that can be used to determine several HMs in various solid samples, but only in small samples [115]. Thus, it can be said that X-ray fluorescence spectrometry is an ideal technique, especially in environmental samples [116], forensic toxicology, for rapid, simultaneous, quantitative mapping of metalloids and metals/trace elements in very low concentrations (even traces) in various human tissues (hard tissues, bones, nails, and teeth) with reasonable specificity, sensitivity, and resolution (Figure 11) [117]. The XRF technique has the ability to simultaneously detect trace elements in very low concentrations, the preparation of the sample for analysis is simple, and regarding the origin of the sample (human nature), the size is limited [115]. However, this method has some drawbacks, such as: the technique/equipment requires the use of a radiation source, LOD depending on sample matrix and specific elements, and for a blood sample it is necessary to identify the parameters that could affect the analysis (depositing the blood sample on the reflector) [118,119].

### 7.3. Atomic Absorption Spectrometry

AAS is a modern analytical method widely used for high sensitivity determinations in various fields, such as: the pharmaceutical industry, water and environmental quality control, clinical and forensic toxicology when involving suspicions of HM poisoning. This method makes it possible to determine concentrations of HMs by the order of ppm or even ppb (using a graphite furnace AAS) [86]. As opposed to classical photometric methods, this method is much more accurate; the analysis is performed in aqueous or organic solutions and does not require a high consumption of reagents. It is a recognized method for qualitative and quantitative determinations of HMs, with a high resolution and a relatively simple way of working [120,121,122]. AAS cannot simultaneously analyze numerous elements, as the light source bulb must be changed while determining the elements and fails to identify elements with resonance lines in the ultraviolet (UV) vacuum area. Also, complex sample matrices might cause interference issues that are difficult to resolve. All of these are shortcomings [123].

### 7.4. Inductively Coupled Plasma Mass Spectrometry

ICP-MS is a type of analytical technique, which uses an inductively coupled plasma to ionize the sample. By atomizing the sample, it creates small polyatomic and atomic ions, which are then detected. It is used and known for its ability to detect metals and non-metals in liquid samples at very low concentrations [124]. ICP-MS is currently the most recent and widely used method for the detection of HMs and metalloids in traces/ultra-traces [125] with a lot of applications in the chemical industry, food processing, environmental analysis, and medical analysis [126]. Emerging since the 1980s and used as a method for the analysis of various multi-element clinical samples, ICP-MS has been increasingly developed for the detection and quantification of metals and metalloids, such as: As, Se, and Pb, etc. [127]. It is considered the gold analytical technique used for the determination of trace elements in biological specimens [128]. This is a superior HM detection method, preferable in comparison to other methods (AAS), because of its high detection speed, accuracy, flexibility, simplicity, and reproducibility. It allows the detection of several elements of the periodic table with an atomic mass ranging from 7 to 250, i.e., from Li to Uranium(U); allowing detection in parts per trillion (ppt) or nanogram/liter concentrations, and can detect and measure variations in isotopic compositions in environmental and geological samples [129,130]. ICP-MS is generally unable to directly identify minor metal ions in samples from the environment. The interaction of the samples’ matrix and decreased concentration metal ions causes ICP-MS’s direct determination to be extremely challenging. Preconcentration and separation are important to increase the sensitivity of trace metal detection [131,132].

ICP-MS is frequently used in the laboratories of pharmacology, clinical and forensic toxicology for quantitative analysis [118,133], and biochemistry, aiming to monitor occupationally exposed people, those with different metal allergies or to identify different HMs or metalloids that may cause intoxication [134]. It also allows the simultaneous analysis of approximatively 30 elements in a single detection, from multiple biological matrices (blood, urine, plasma, hair, nails, tissues, and organs), resulting in an optimal gain in sensitivity [127]. Performing the analysis involves a high cost as the samples need to be pre-dissolved into solutions. Due to low salt tolerance, the limits of detection can really be up to 50 times worse. Several light elements cause significant interference in ICP-MS, such as Ca, S, K, Fe, and Se [121].

Table 8 presents the most modern analytical techniques used for HM determination in biological samples.

### 7.5. Hyphenated Techniques and Speciation Analytics

Hyphenated analytical techniques include approaches that combine chromatography with spectroscopic or spectrometric methods to analyze a variety of biochemical or toxicological compounds. Furthermore, these techniques are generally preferred, especially in trace analysis, since they greatly limit the danger of cross-contamination due to sample manipulation. Additionally, the fraction of interest cannot typically be transferred quantitatively using off-line procedures [135,136].

Over time, the beneficial effects of combining spectroscopic or spectrometric techniques with separation methods for the quantitative and qualitative assessment of unidentified chemicals in complex fractions and extracts has been established. Spectroscopic or spectrometric detection techniques, such as mass spectrometry (MS), Fourier transform infrared (FTIR), ultraviolet-visible (UV-Vis) absorbance or fluorescence emission, nuclear magnetic resonance spectroscopy (NMR), and photodiode array (PDA), are linked to liquid chromatography (LC), typically high-performance liquid chromatography (HPLC), capillary electrophoresis (CE), or gas chromatography (GC), to obtain detailed information on the structure, leading to the detection of different compounds, including HMs, present in various biological samples [137].

Analytical techniques for detecting and speciating metals and metalloids have drawn considerable scientific interest because of their significant environmental and public health impacts [138].

Speciation analytics refers to the assessment of different chemical and physical forms of a specific element. The concept of speciation can be used to identify the presence of several configurations of a certain analyte in the sample under observation, such as the attachment to various ligands or the presence of various oxidation states. Moreover, selectivity in order to ensure the compounds of interest are identified and sensitivity in order to accurately assess the concentration of the analyte in the sample are specific parameters required for an appropriate speciation analysis [139].

The distinction between physical and chemical speciation is also feasible. Chemical speciation can be divided into distribution speciation involving the exploring or evaluating of particular chemical structures and identifying particular chemical forms in particular elements of the samples and screening speciation that involves seeking and ascertaining selected chemical structures [140,141]. According to the outcomes of toxicological testing, a specific entity’s proportion in a mixture often has a more significant impact on living organisms than its overall concentration [139].

The inorganic ions of As, Se, Hg, Pb, and Sn, whose toxicological characteristics are varied, are those for which speciation analyses are most frequently utilized. As speciation is a topic that is constantly evolving due to advancements in analytical methods and growing toxicological knowledge. As speciation is not frequently provided as an examination in clinical laboratories, despite the fact that speciation procedures have been widely developed. Furthermore, HPLC-ICP-MS is typically used to perform As speciation on urine and blood samples. Micro-liquid chromatography coupled to an ICP-MS spectrometer (μLC-ICP-MS) is an experimental approach established as a novel and quick analytical method for determining the different forms of As [139,142].

Another experimental study assessed the concentration of Se in human serum by also applying hyphenated techniques. After an enzymatic hydrolysis, the resulting compound (i.e., selenomethionine) was evaluated using reversed phase-HPLC (RP-HPLC), combined with collision/reaction cell inductively coupled plasma-quadrupole mass spectrometry (CRC ICP-QMS) [143].

The detection of Hg, Pb, and Sn species in urine samples has been conducted by applying a GC/MS-MS method. Capillary GC combined with an ion-trap mass spectrometer with electron impact ionization in the tandem-MS mode (MS-MS) was used to separate and identify the toxic metallic ions. Furthermore, for increased accuracy, a very efficient method of sample preparation was established, focusing on chemical modifications with sodium tetraethylborate and subsequent headspace solid phase microextraction [144].

The hyphenated techniques broaden the novel and extend the prospects. Their primary benefits are extremely low detection and measurement thresholds, a negligible impact of external interference on the analytical assay, and extremely high accuracy and repeatability of outputs [139]. The disadvantages and expensive price of the equipment are undoubtedly the limits of the hyphenated approaches. Since they are difficult to acquire, laboratories do not frequently use them. Additionally, the use of hyphenated procedures needs a thorough understanding of the analytical techniques and equipment. Rather than being used for the usual analyses, these devices are exceedingly expensive and are only used in research. However, the hyphenated strategies have been evolving and becoming more and more significant, which is supported by the growing body of publications on the topic and by new methods/techniques [139,145].

## 8. Conclusions and Future Perspectives

The potential of HMs to damage DNA and membranes, as well as to interfere with the activity of proteins and enzymes has demonstrated that they represent a significant risk to human health.

At the present moment, AAS is the dominant analytical technique for the detection of trace elements (metals, and oligo-elements) in the human body, and we are witnessing a more and more evident shift from flame-electro thermal atomic spectrometry to ICP-MS, a more modern and effective method. However, the choice of the analytical method used for the detection of the different metals will remain at the discretion of the toxicologist/chemist who considers the metals to be detected, their number, the analyzed matrix (biological samples), the concentration of the analyte in the matrix, and the intended purpose (toxicological/nutritional screening, therapeutic purpose, and prevention, etc.).

TMA is of great importance for monitoring the health of the population, not only for studying the effects of occupational exposure, but also for health in general, by determining both nontoxic minerals in the human body and toxic minerals (HMs). Although some authors claim that TMA is an alternative method of investigation or even a fraud (some considered TMA to be “unscientific, economically wasteful and probably illegal”), its importance cannot be denied [146]. In 2011, a publication emerged in the scientific literature that clarified to some extent the status of TMA. This diagnostic method cannot accurately, clearly, and realistically determine what causes an abnormal concentration of minerals in the hair. However, it can be stated that the results of the TMA, in conjunction with laboratory biochemical analyses (i.e., determination of δ-aminolevulinic acid (ALA), coproporphyria/Pb poisoning, or determination of As acute hemolytic anemia, the dark color of urine, detection of plasma Hg, coproporphyria or DMPS challenge test etc.) can lead to an accurate diagnosis in terms of the disorders that the patient suffers from, possible intoxication or possible procedures that the patient undergoes [22].

In the case of an HM detection in forensic practice, it is extremely important to collect as many biological fluids, tissues, and organs as possible, to process them as quickly as possible and to analyze them with modern high-performance equipment. All of these factors, together with a histopathological examination and an investigation into possible diseases and habitual behaviors, can lead to the most accurate determination of the cause of death.

Hyphenated techniques, which combine multimodal detectors with chromatographic separative methods, are becoming effective substitutes with potential use in the evaluation of HMs.

Given the fact that HMs are toxic elements for both human health and the environment, and due to the high degree of bioaccumulation in the human body, it would be advisable to monitor them continuously. Nowadays, due to the modernization of laboratories, the development of biological methods of identification and quantification, using biomarkers it is possible to establish certain criteria and standards that would help protect human health and the environment from HM contamination. TMA, combined with high-performance laboratory analysis, can be considered a vital barometer of the population’s health, as it creates a true mapping of all the biochemical reactions in the body, which through their disturbances can trigger various diseases or even intoxications.

## Figures and Tables

**Figure 4:**
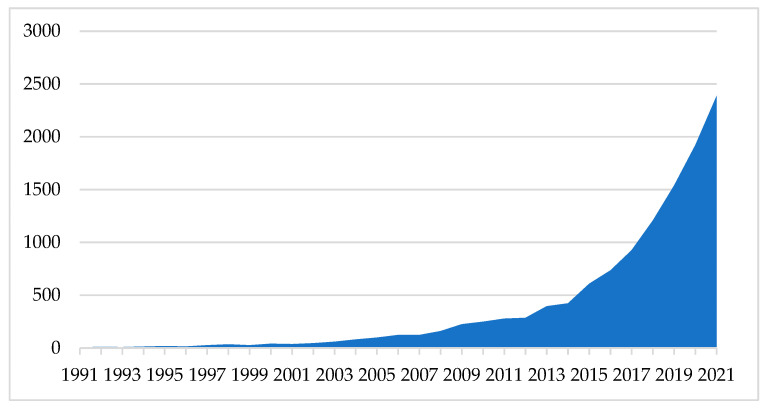
Number of documents published/year.

**Table 1:** The top-10 keywords for each period based on the number of occurrences.

Word/Term	Average Citation/ Article	Occurrence	Word/Term	Average Citation/ Article	Occurrence
	1975–2010			2011-to Present	
Heavy metals	84.15	361	Heavy metals	17.07	3920
Cadmium	83.94	302	Cadmium	20.05	1693
Lead	90.56	252	Contamination	21.77	1628
Heavy metal contamination	84.84	138	Pollution	18.78	1514
Exposure	83.29	128	Lead	19.68	1345
Zinc	85.05	127	Trace-elements	23.02	1127
Mercury	101.54	112	Accumulation	18.39	1074
Accumulation	53.28	102	Exposure	20.78	997
Pollution	54.79	101	Soil	16.41	994
Soil	85.51	95	Water	16.73	915

**Table 2:** Top-10 countries according to the number of articles published.

Country	Articles No.	Citations No.	Average Citation/Document	Total Link Strength
China	3944	106,114	26.91	1400
United States	1346	52,942	39.33	971
India	1157	29,987	25.92	513
Pakistan	647	20,070	31.02	629
Iran	617	11,328	18.36	286
Italy	596	17,895	30.03	278
Spain	477	14,915	31.27	256
Turkey	447	7222	16.16	141
Australia	424	14,624	34.49	475
Brazil	377	5006	13.28	162

**Table 3:** Top-five most-cited documents.

Author (Year)	Title of the Article	Journal	IF	Citations	Ref.
Jarup (2003)	Hazards of heavy metal contamination	*British Medical Bulletin*	4.291	3507	[4]
Kampa (2008)	Human health effects of air pollution	*Environmental Pollution*	8.071	2188	[11]
Ali (2013)	Phytoremediation of heavy metals–Concepts and applications	*Chemosphere*	7.086	2118	[12]
Salt (1998)	Phytoremediation	*Annual Review of Plant* *Biology*	22.192	1663	[13]
Li (2014)	A review of soil heavy metal pollution from mines in China: Pollution and health risk assessment	*Science of the Total* *Environment*	7.963	1523	[14]

**Table 4:** Top-10 journals according to the number of articles published.

Source	Documents No.	Total Citations	Average Citation/Article	Impact Factor	Publisher
*Environmental Science* *and Pollution Research*	750	10,916	14.55	4.223	Springer
*Science of the Total Environment*	630	33,435	53.07	7.963	Elsevier
*Chemosphere*	409	16,734	40.91	7.086	Elsevier
*Environmental Monitoring* *and Assessment*	333	6538	19.63	2.513	Springer
*Ecotoxicology and* *Environmental Safety*	309	10,759	34.82	6.291	Elsevier
*Environmental Pollution*	305	15,755	51.66	8.071	Elsevier
*International Journal of Environmental Research and Public Health*	291	4481	15.40	3.39	MDPI
*Environmental Geochemistry* *and Health*	290	4829	16.65	4.609	Springer
*Journal of Hazardous Materials*	188	7919	42.12	10.588	Elsevier
*Human and Ecological* *Risk Assessment*	177	2541	14.36	5.19	Taylor and Francis Ltd.

Legend: MDPI, Multidisciplinary Digital Publishing Institute.

**Table 5:** The toxic effects of heavy metals on different systems.

Toxicity Effects Summary	Heavy Metals	Refs.
**Central nervous system (CNS)**		
Brain injuries: − adults: headache, fatigue, hallucinations, paralysis, antisocial behavior, trouble thinking, loss appetite, and allergies; − children: decrease in attention interval, mental retardation, autism, ↓IQ, lower educational level, and encephalopathy; Multitude of cerebellum lesions; Sensory disturbances, loss of peripheral vision, gait disturbance, incoordination, hearing, and speech impairment; Trembling, sleeplessness, tugging, weakness, headaches, and muscular atrophy; Mercury poisoning: “Hatter’s Shakes” Syndrome; Neurodegenerative diseases: Parkinson’s, Alzheimer’s, Lou Gehrig‘s, dialysis dementia and neurotoxicity; nervousness, somnolence, memory loss, and intellectual disability; Cyanosis, toxically polyneuropathy, and paresis.	Pb, MeHg, Hg, Al, As	[18,19,22,23,24,25,26,27,28,29]
**Respiratory system**		
Pulmonary fibrosis, interstitial and granulomatous pneumonia, asthma, pulmonary edema; Pulmonary edema, asthma, and tuberculosis; Burning pain in the chest, cough, dyspnea, fibrosis, pneumonia, and pulmonary edema; Obstructive pulmonary disease, and asthma.	Al, As, Hg, Pb	[28,30,31]
**Cardiovascular system**		
High blood pressure, oxidative stress, fatigue, and high risk of cardiovascular accident; Cardiovascular lesion, toxic myocarditis, dysfunction and inflammation of the myocardium, and congenital heart defects; Myocardial injuries: cardiomyopathy, low blood pressure, cardiac arrhythmia, and heart failure.	Pb, Al, As	[29,30,32,33,34]
**Skeletal system**		
Most lead is stored in the bones: − calcium, magnesium, iron deficiency is exacerbated; − bone-to-blood mobilization; − inhibiting the renal 1-λ-hydroxylase enzyme; Low-bone remodeling, impaired formation of calcium, fluoride, phosphorus → rickets, osteoporosis, osteomalacia, and decreased bone density.	Pb, Al	[18,25,30,35,36]
**Gastrointestinal system**		
Abdominal and gastric dysfunction: loss appetite, abdominal pain, weakness, vomiting, diarrhea, constipation, and metallic taste in the mouth; Excessive intestinal inflammation, and damage to the intestinal microbiota; Metallic taste in mouth, garlic-smelling breath, gastric ulcers, heartburns, nausea, vomiting, abdominal pain, and bloody rice water diarrhea.	Pb, Al, As	[18,25,29,30,34]
**Hepatorenal system**		
Liver effects, disturbance of normal kidney function (proteinuria); Hepatic lesions, oxidative injuries → necrosis, tissue degeneration, and biochemical derangement; Metallic taste in the mouth.	Hg, Al, As	[29,30,37]
**Reproductive system**		
In women: high risk of miscarriage, low birth weight, stillbirth, and children—developmental problems; In men: impotency, sterility, reduction in sperm count and motility; Spontaneous abortion, birth defects, miscarriage, and low birth weight.	Pb, As	[25,29]
**Hematopoietic system**		
Adversely effects the metabolism of blood cell and blood, heme synthesis is disturbed; Side effects of red blood cell metabolism, anemia, and leukopenia; Modifies blood-biochemical parameters, anemia, “metal fume fever” syndrome → fever, chills, fatigue, and elevated leukocyte count.	Pb, As, Al, Hg	[18,22,25,30]
**Dermal**		
Skin lesions: skin depigmentation/nuances, “rain drops on a dusty road” Syndrome (keratosis), alopecia, over-exfoliation of the skin on the extremities, and dermatitis.	As	[18,29]

**Table 6:** Binding biological limit values concerning occupational exposure.

Substances	Biological Indicator	Biological Matrix	Sample Collection	BBLV
Aluminium	Aluminium	Urine	At the end of the shift	200 μg/L
Arsenic and AsH_3_	Arsenic	Urine	At the end of the week	50 μg/gC
		Hair	At the end of the week	0,5 mg/100 g
Mercury and its compounds	Mercury	Blood	At the end of the shift	10 μg/L
	Mercury	Urine	At the beginning of the next shift	30 μg/gC
Lead	Lead	Blood	At the end of the shift	70 μg/100 mL
	Lead	Urine	At the end of the shift	150μg/L
	Lead	Hair	At the end of the shift	3 mg/cm
	δ-aminolevulinic acid	Urine	At the end of the shift	10 mg/L
	Coproporphyrins	Urine	At the end of the shift	300 μg/L
	Free Erythrocyte protoporphyrin	Blood	At the end of the shift	100 μg/100 mL erythocytes
Lead tetraethyl	Diethyl Lead	Urine	At the end of the shift	25 μg/L
	Total Lead	Urine	At the end of the shift	50 μg/L

**Table 7:** Advantages and disadvantages of different biological matrices used to identify heavy metals (HMs).

Advantages	Disadvantages	Refs.
**Blood**		
The most-used biological matrix; Releases information about HM at the present moment; Preferable to identify Pb and Hg salts; Short residence times 2–3 h or 3–4 days.	Invasive sampling method; Does not show the cumulative degree of exposure; Risk of contamination during collecting and storage; Requires cold storage; Blood levels are transient, independent of those from the tissues; The anticoagulant may interfere with the method of determination; Very low concentrations cannot be detectable by common analytical methods.	[65,87,88,89]
**Human urine**		
Shows how the HM is excreted at the time of collecting; Simple/easy method of collecting; Preferable for the determination of certain HMs: inorganic forms of Hg and As; Accessible and available in large Volumes.	Provides information only on relatively recent exposure; Risk of contamination during collecting and storage; If the sample is not processed immediately, it must be frozen.	[87,89,90]
**Vitreous humor/Retinal tissues**		
High affinity for HM; Shows the cumulative degree of exposure.	Difficult to collect (preferably from dead bodies); High risk of contamination during collecting and storage; Available in small quantities (2–4 mL).	[78,89,91,92]
**Hair**		
The most important biomarker for health assessment; Provides information on long-term exposure time (from few weeks to years); Simple, frank, and non-invasive method of collecting; Shows the direct correlation between hair analysis and various diseases.	Some elements may be deposited by different cosmetic procedures (dyeing, blenching, permanent waving, and smoke); Seasonal variations; The lack of reference ranges for the interpretation of results.	[65,68,93,94]
**Nails**		
Shows long-term exposure and intake of HM; Easy to collect, transport, store and prepare for analysis; It does not require a large sample; Toenails have been used as a biomarker in forensic, clinical, and environmental Studies.	Can be easily contaminated with some medications or nail polish; Small nail amount → low detection limit; Differences in the analysis of the toenails and fingernails; Use of nails as a biomarker for some elements is not well characterized.	[95,96]
**Bones**		
Bioindicator of a long-term exposure; Accumulates HMs with a high affinity; In particular, femurs and tibiae can fix HM very well; Chemically stable; Ideal calcified tissues for long-term Exposure.	Not very accessible for sampling and measurement; The mineral phase is subject to turnover; Release and fluctuation of different minerals from the bones to blood; Significant differences in HM concentrations between different bones.	[25,87,97]
**Human teeth**		
Provides a cumulative and permanent record of recent/past exposure to HMs; Dentine and enamel also suitable as bioindicators of exposure; Offer several advantages over other bioindicators of exposure (liver, and kidney); Readily accessible materials; Low-rate elimination of HM.	Primary teeth may be used as a bioindicator of long-term exposure, but they have a short lifespan; Use the non-carious teeth toreduce the confounding factors; The chemical composition of the teeth may change after the influence of some factors (drugs, food, and medicines); The extraction of permanent teeth is a problem.	[82,83,84,97,98]
**Human organs/tissues**		
Kidney, brain, lung, liver, heart, and muscles are targeted tissues for HM deposit.	Difficult sampling.	[25,92,99]

**Table 8:** Modern analytical methods used for HM determination in biological samples.

Analytical Methods	Biological Matrix	Limit of Detection (LOD)	Applications
LTT MELISA HMs	Blood	SI < 2, NEGATIVE, there is no type IV, sensitization for tested metals; SI = 2–3, HIGHLY suggestive result for a POSITIVE reaction SI > 3, POSITIVE	Metal allergies
X-ray Fluorescence	Blood, soft tissues, bones, organs, and hair	0.1–1 mg/g	Clinical, forensic toxicology Occupational exposure
AAS	Blood, urine, and vitreous humor; Soft tissues; Hair, bones, nails, organs (liver, lung, kidney, brain, and skin).	0.001–0.5 μg/L	Clinical and forensic toxicology Occupational exposure Acute/chronic intoxications
ICP-MS	Blood, urine, and serum; Vitreous humour, soft tissues, hair, nails, and bones; Organs (liver, lung, kidney, brain, and skin).	0.001–0.1 μg/L	Clinical and forensic toxicology Occupational exposure Acute or chronic intoxications

LTT, HMs test-Lymphoblastic Transformation Test; AAS, Atomic Absorption Spectrometry; ICP-MS, Inductively Coupled Plasma Mass Spectrometry; and SI, stimulation index.

## Data Availability

Not applicable.
